# 

**Published:** 1895-01

**Authors:** 


### Southern Medical Record.

A Monthly Journal of Medicine and Surgery.

VOL. XXV. ATLANTA, GA., JANUARY, 1895. No. 1.

EDITORS:

[illegible]. W. GRIGGS, M. D. | WILLIS F. WESTMORELAND, M. D.

J. McFADDEN GASTON, M. D. | LOUIS H. JONES, M. D.

DAN. H. HOWELL, M. D., BUSINESS MANAGER.

WM. S. GOLDSMITH, M. D., MANAGING EDITOR.

**TERMS: $2.00 Per Annum; Single Copy, 20 Cents.**

**Contents on Page 5.**

**PUBLISHED ON THE FIFTEENTH OF EACH MONTH.**

Entered at the Post-office at Atlanta, Ga., as Second-Class Matter.

The Foote & Davies Co., Atlanta, Ga.

